# Relationship between lifestyle: physical activity, diet, smoking, and the duration of symptoms of SARS-CoV-2 virus infection

**DOI:** 10.1186/s12879-025-11008-z

**Published:** 2025-12-09

**Authors:** Jarosław Rachuna, Łukasz Madej, Elżbieta Zawierucha, Marcin Zawierucha, Syliwa Knap, Drążkiewicz Anna, Kacper Szafraniec, Ewa Kowalska, Monika Wawszczak-Kasza, Anna Goliszek, Dariusz Mika, Piotr Lewitowicz, Krzysztof Ołownia

**Affiliations:** 1 Department of Medical Genetics and Laboratory Diagnostics, Collegium Medicum, Jan Kochanowski University, Kielce, 25-317 Poland; 2 Faculty of Medical and Health Sciences, Casmir Pulaski Radom University, Bolesława Chrobrego 27, Radom, 26-600 Poland; 3 Regional Science and Technology Center, Podzamcze 45, Chęciny, 26-060 Poland; 4 Department of Neurology, Institute of Medical Sciences, Jan Kochanowski University in Kielce, IX Wieków Kielc 19A, Kielce, 25-317 Poland; 5 Department of Agriculture and Rural Development, The Marshal Office of the Świętokrzyskie Voivodeship, Al. IX Wieków Kielc 3, Kielce, 25-516 Poland; 6 Collegium Medicum, Jan Kochanowski University in Kielce, Jan Kochanowski University, Kielce, 25-317 Poland

**Keywords:** SARS-CoV-2, Virus, Infection, COVID-2019, Lifestyle, Diet

## Abstract

In this study, patients infected with severe acute respiratory syndrome coronavirus 2/coronavirus 2019 (SARS-CoV-2/COVID-2019) had their diets, levels of physical activity, and smoking habits evaluated, along with the effects of these parameters on disease symptom duration. The study followed a cross-sectional design. Data were collected from a group of SARS-CoV-2-infected patients who were tested at the Regional Science and Technology Center from January to December 2021. The survey research included a cohort of 3,155 SARS-CoV-2 patients. Nasopharyngeal swab samples were tested using reverse transcription polymerase chain reaction (RT-PCR), confirming infection by detecting the presence of SARS-CoV-2 RNA. The data did not identify any potentially harmful effects of cigarette smoking on the duration of COVID-19 symptoms. It was confirmed that a high-calorie diet was associated with a reduction or absence of disease symptoms. The results confirmed that physical activity positively affected the duration of COVID-19 symptoms by shortening it (*p* ≤ 0.05). The data demonstrated a relationship between cigarette smoking, diet, physical activity, and the severity of symptoms during COVID-19 infection.

## Background

The COVID-19 pandemic, precipitated by the SARS-CoV-2 virus, underscores the imperative for a comprehensive understanding of factors influencing the duration of symptoms. Lifestyle choices, including physical activity, diet, and smoking, have been implicated in modulating viral infections. This study explores their association with the duration of SARS-CoV-2 symptoms, aiming to provide insights for enhanced management strategies.

The investigation focuses on how lifestyle factors—physical activity, diet, and smoking—affect symptom duration among individuals infected with SARS-CoV-2. By elucidating these relationships, the study aims to offer valuable insights for optimizing management approaches. Existing literature suggests that regular physical activity and a balanced diet may strengthen immune function, potentially influencing the trajectory of viral infections. Conversely, smoking has been linked to heightened susceptibility to respiratory infections and worsened COVID-19 outcomes.

Despite advancements, the precise impact of lifestyle factors on symptom duration remains ambiguous. Conflicting findings necessitate further exploration of these relationships and their underlying mechanisms to guide interventions. This study aims to bridge existing knowledge gaps by examining how lifestyle choices influence the duration of COVID-19 symptoms, with the objective of informing public health policies and encouraging individuals to adopt behaviors conducive to mitigating the impact of SARS-CoV-2 infection.

## Introduction

It seems that the main threat to public health in the world may be newly emerging viral diseases [1]. Over the past few years, new outbreaks of new viruses, including severe acute respiratory syndrome corona virus 1 (SARS-CoV 1) in 2002, H1N1 influenza (2009), Middle Eastern respiratory syndrome corona virus (MERS-CoV) in 2012, Ebola (2013), and Zika virus (2015) [1–5]. December 2019, in the Chinese capital of Hubei province, Wuhan, the first cases of patients with a previously unidentified pathogen were recorded. A strain of SARS-CoV-2 coronavirus unprecedented in humans has been identified as the causative pathogen [6–7]. The World Health Organization (WHO) officially declared a state of pandemic in the first half March 2020 [8]. The SARS-CoV-2 coronavirus is an enveloped virion of circular shape with positive single-stranded RNA. The length of the genome is between 26 and 32kb [9].

A distinctive feature of the virus is the occurrence of protein protrusions on the surface. An S-spike glycoprotein is responsible for attaching the virus to host cell receptors, including primarily the human angiotensin converting receptor (ACE2) [6, 10]. The high genetic variability of the SARS-CoV-2 virus, mainly in the area of the gene encoding protein S, contributed to the emergence of new mutations of this strain. The most epidemiologically significant variants are alpha, beta, gamma, delta, and omicron [11, 12]. COVID-19 infection mainly affects the upper and lower respiratory tracts. Adults infected with SARS-CoV-2 are most likely to experience mild to moderate respiratory symptoms, such as cough and shortness of breath or sore throat [12]. Clinical improvement in mild to moderate cases usually occurs about 10 days after the onset of the first symptoms [10]. Other symptoms that can be observed over the course of the disease are loss of smell and taste, headache, fever, diarrhea, rash, and eye irritations [12]. Some patients may develop serious complications leading to acute respiratory failure, interstitial pneumonia caused by severe restrictive insufficiency due to fibrosis or shock, and in some cases, death [13, 14]. The intensity of symptoms during illness depends on many factors, namely, the general health of the patient, his/her age, and/or comorbid diseases [15]. Despite many efforts to curb the pandemic, the virus continues to be present in people in many countries with varying degrees of severity of clinical manifestations. Research into risk factors is still ongoing, which could help reduce the number of deaths and hospitalized people. Moreover, the COVID-19 pandemic may affect the change in our lifestyle via long-term limitations on various activities [16]. Literature reports do not clearly indicate whether cigarette smoking significantly affects the morbidity and course of COVID-19. To date, not enough data to clearly state what impact smoking has on the course of COVID-19 disease are available. Another factor that undoubtedly affects overall health is physical activity. Some evidence exists in which it has been shown that physical activity positively affects human health. According to WHO, physical activity is any movement of the body, caused by the action of skeletal muscle, requiring energy expenditure. Therefore, physical activity is not only considered a professional sport but also all forms of movement, such as walking, dancing, or housework [17]. In addition, physical activity has a beneficial effect on cardiovascular health via an increase in muscle strength, lung capacity, and improving mental health. Thanks to these mechanisms, regular physical activity can play a role not only in reducing the incidence of many chronic diseases, but also in mitigating the COVID-19 pandemic [18]. Physical activity has declined significantly during the pandemic, while eating habits have remained essentially unchanged [19]. Even before the outbreak of the pandemic, insufficient physical activity by the population was described as a global problem [20]. Biochemical changes occurring in the body during physical activity seem to have a positive effect also on the regeneration of the body during the COVID-19 infection. The intensification of the phenomenon of reduced activity during the pandemic is not the only problem. Staying locked at home, we reached more often for unhealthy snacks, which caused overeating and consequently generated a positive energy balance. In addition, increased alcohol consumption was observed. Viral infections expose the body to significant energy expenditure, so the consequences of such a viral infection largely depend on the nutritional status. To overcome infection, a balanced gut microbiome is essential for keeping the immune system working properly. The intestinal microbiota avoids an excessive immune response that can lead to tissue damage. Both an unhealthy diet and lack of exercise (leading to obesity) combined with malnutrition are the main causes of changes in the functioning of the immune system. Thus, they have a destructive effect on the body’s immunity, increasing greater susceptibility to viral and bacterial infections [21, 22]. In conclusion, the factors mentioned earlier may therefore have a potential impact on the likelihood of infection and the severity of the severe course of COVID-19 disease.

The main objective of the study was to evaluate the contribution of various aspects of patients’ lifestyles, such as diet, physical activity and smoking, to the period of COVID-19 symptoms.

## Materials and methods

### Patients

In the present study, data were collected from 3155 patients who presented to Regional Sciences and Technology Center from January to December 2021 with positive SARS-CoV-2 tests. The questionnaire was administered via telephone between one week and three months after the patient received a positive test result, with the timing depending on various factors.

A total of 3155 participants took part in the project. Infection caused by the SARS-CoV-2 virus was confirmed by testing the presence of SARS-CoV-2 virus RNA in nasopharyngeal swab samples using the reverse transcriptase polymerase chain reaction (RT-PCR) method virella SARS-CoV-2 seqc real time RT-PCR Kit (Gerbion GmbH & Co. KG Germany). Transport and insulation of the material was carried out in accordance with the principles of good laboratory practice. The isolated material was subjected to RT-PCR detect the viral RNA from SARS-CoV-2. Tests recommended for diagnostics use detection of at least two viral genes.

The group of patients who indicated the absence of any symptoms but received a positive result for coronavirus were tested as part of routine testing after contact with an infected self, or the patients came in for testing to obtain the PCR result required for travel or other reasons.

### Data collection

Initially, primary data, including demographic and laboratory data, were collected from medical records of all patients. Next, further information was obtained using phone-survey. First, patients consented to participate in the project and then completed the questionnaires by answering the questions asked by the interviewer. When collecting data, RCNT employees used a specially prepared questionnaire entitled medical questionnaire for a study participant, which included several questions about lifestyle. The author’s questionnaire was composed of a set of 16 questions, which patients answered voluntarily and anonymously. The questions contained in the form addressed the intensity of symptoms of COVID-19 disease, the duration of symptoms, and general body condition and lifestyle (diet, smoking, physical activity) among others. All patients were classified on the basis of symptoms ranging from absence of symptoms to those persisting even more than 10 days. The occurrence of symptoms was divided into groups: no symptoms, 1–5 days of persistence of symptom, 6–10 days of persistence of symptoms, and presence of symptoms for more than 10 days. Answers to diet-related questions were used to divided patients into several categories. Among the respondents it was possible to distinguish vegetarians, people who eat only small amounts of meat, and those who often reach for meat dishes. The subjects also reported the number of vegetables and fruits consumed and the amount of water drunk during the day. Among the questions asked were also those regarding frequent consumption of warm soups, pickled products, fast food, sweets, and low- and high-calorie meals. Questions about the frequency of physical activities were also considered. Respondents answered the question about how often they undertake physical activity, choosing from the answers: (1) less than once a month, (2) once a month, (3) several times a month, (4) once a week, (5) several times a week, or (6) every day. This breakdown of physical activity was used because patients reported a single activity once every two or three weeks. In addition, the respondents subjectively assessed the level of their own physical fitness as very good, good, average, or low.

Patients were also asked questions about cigarette smoking, thus dividing them into different groups: (1) smokers, (2) never smokers, (3) stopped smoking in the last 10 years, or (4) people who stopped smoking more than 10 years ago. .

### Statistical data analysist

The data were entered into an Excel spreadsheet (Microsoft, USA) and then exported to a statistical program *Statistica* (TIBCO Software Inc., USA). Several methods of statistical analysis were used for the statistical description of numerical variables, mean, standard deviation, minima and maxima were chosen. For the statistical description of qualitative variables, abundance and percentages. For the analysis of differences between two groups. Student’s t-test was used. For the analysis of interactions of qualitative variables, a chi-squared test was chosen, and for the analysis of correlation between the variables, the r-Pearson’s and Spearman’s rho correlation coefficients were used.

The inclusion criteria included positive results from a real time reverse transcriptase polymerase chain reaction (qRT-PCR) assay of nasopharyngeal swab specimens and full data were available.

## Results

The team of researchers found a relationship between diet, physical activity, smoking and symptoms of SARS-CoV-2 infection. Most respondents reported that they lived in the city 54.99%, rural residents accounted for 43.43%, while 1.58% of participants did not report their residence. Among the surveyed people, 1545 women (48.97%) and 1557 men (49.35%) and 53 (1.68%) people who did not determine their gender were included. No statistical difference between sex and the occurrence of symptoms was found. It was determined that among women, symptoms persisted statistically significantly longer than in men (< 10 days). The largest percentage of infected people were between 30 and 50 years.

The research team identified a relationship between diet, physical activity, smoking, and symptoms of SARS-CoV-2 infection. In the study, data were collected from 3,155 patients who presented to the Regional Science and Technology Center (RCNT) from January to December 2021 with positive SARS-CoV-2 tests. Most respondents reported living in urban areas (54.99%), while rural residents accounted for 43.43%, and 1.58% of participants did not specify their place of residence. Among the surveyed individuals, there were 1,545 women (48.97%), 1,557 men (49.35%), and 53 people (1.68%) who did not specify their gender. No statistical difference between gender and the occurrence of symptoms was found. However, it was determined that symptoms persisted statistically significantly longer among women than men (lasting less than 10 days). The largest percentage of infected individuals were between the ages of 30 and 50 (Tables 1, 2, 3 and 4).

**Table 1:** Participants’ demographic and characteristic details

		Severity				Duration		
**Demographic Data**		Total	NO SYMPTOMS	SYMPTOMS	p-value	< 10 days	> 10 days	p-value
		*n* = 3155 (100%)	*n* = 487 (15.4%)	*N* = 2668 (84.56%)		1711/2668 (64.13%)	957/2668 (35.87%)	
**Sex**	Female N (%)	1545 (48.97)	216/1545 (13.98)	1329/1545 (86.02)	NS	786/1329 (59.14)	543/1329 (35.15)	0.00001
	Male N (%)	1557 (49.35)	252/1557 (16.18)	1305/1557 (83.82)		901/1305 (57.87)	404/1305 (30.96)	
	Not determinate (%)	53 (1.58)	12/53 (22.64)	41/53 (77.36)		29/41 (70.73)	12/41 (29.27)	
**Residence**	City N (%)	1735 (54.99)	258/1735 (14.87)	1477/1735 (85.13)	NS	925/1477 (62.63)	552/1477 (37.37)	0.035657
	Village N (%)	1370 (43.42)	214/1370 (15.62)	1156/1370 (84.38)		758/1156 (65.57)	398/1156 (34.43)	
	No data (%)	50 (1.58)	7/50 (14.00)	43/50 (86.00)		34/43 (79.07)	9/43 (20.93)	

Significance level, 0.05, NS-not significant

**Table 2:** The impact of cigarette smoking on the duration of covid-19 symptoms

Smoking	1–5 Days *N* (%)	6–10 Days *N* (%)	> 10 Days *N* (%)	NO SYMPTOMS *N* (%)
They never smoked *N* = 2352	540 (23.0)	745 (31.7)	744 (31.6)	**323 (13.7)**
Did not smoke ≤ 10 years *N* = 224	54 (24.1)	80 (35.7)*	59(26.3)	31(13.8)
They did not smoke > 11 years *N* = 126	17 (13.5)	43 (34.1)	44 (34.9)*	22 (17.5)
Smokers *N* = 441	114(25.9)*	117 (26.5)	109 (24.7)	101 (22.9)*

*- the highest percentage of respondents in a given group

A study conducted between January and December 2021 found that among the study participants who answered a question about smoking (3143 people) 74.8% of people had never smoked 7.1% of persons who have stopped smoking within the last 10 years 4.0% of people who stopped smoking more than 10 years ago and 13.9% of active smokers. The asymptomatic course of COVID-19 disease occurred in only 13.7% of people in the never-smoked group. Non-smokers in 23.0% were ill during the time interval of 1 to 5 days. The vast majority of declared non-smokers had symptoms characteristic of SARS-CoV-2 infection. Among non-smoking study participants, 31.7% reported symptoms lasting from 6 to 10 days, 31.6% of subjects had symptoms lasting more than 10 days. Symptoms of disease duration in people who quit smoking (up to 10 years) most often persisted for up to 10 days (35.7% and over 10 days occurred in 26.3% of the subjects. People in this group of respondents were much less likely to report symptoms up to 5 days (24.1%), while the complete absence of symptoms was noted in the least numerous group (13.8%). Another study group were people who had stopped smoking in the last 10 years and as it turns out, they were ill for a longer time period, and their symptoms were usually present for more than 10 days (34.9%) or up to 10 days (34.1%). Only 13.5% of the subjects (1–5 days) reported a short disease duration, and the complete absence of symptoms was noted in 17.5%. Study participants who admitted to active cigarette smoking struggled with COVID-19 symptoms lasting up to 10 days in 26.5% and more 10 days in 24.7%. In agreement with study results, the infection in smokers lasted up to five days in 25.9% of the subjects, but interestingly it was also often asymptomatic (22.9%). According to our research, the lack of symptoms of COVID-19 disease in the group of smokers was a common phenomenon. Among the respondents, 12 people did not answer this question.

### The effect of diet on the course of COVID-19

**Table 3:** The impact of cigarette smoking on the duration of covid-19 symptoms

DIET	1–5 DAYS *n* = 725 N (%)	6–10 DAYS *n* = 920 N (%)	> 10 DAYS *n* = 954 N (%)	NO SYMPTOMS *n* = 472 N (%)
Balanced	220 (30.3)	285 (29.1)	282 (26.6)	145 (30.7)*
Lots of meat	248 (34.2)*	297 (30.3)	262 (27.5)	151 (32.0)
Little meat	158 (21.8)	261 (26.6)	267 (28.0)*	108 (22.9)
Vegetarianism/Veganism	10 (1.4)	18 (1.8)	19 (2.0)	19 (4.0)*
Lots of fruit and vegetables	329 (45.4)	480 (49.0)*	451 (47.3)	213 (45.1)
Few vegetables and fruits	97 (13.4)	126 (12.9)	122 (12.8)	77 (16.3)*
High Calorie	148 (20.4)*	175 (17.9)	158 (16.6)	96 (20.3)
Low Calorie	143 (19.7)	192 (19.6)	196 (20.5)	119 (25.2)*
Lots of water > 8 glasses per day	361 (49.8)*	466 (47.6)	432 (45.3)	222 (47.0)
Little Water	191 (26.3)	280 (28.6)	303 (31.8)*	150 (31.8)*
Silage	373 (51.4)	530 (54.1)	514 (53.9)	**273 (57**,8)*
Warm Soups	491 (67.7)	685 (69.9)*	684 (71.7)	330 (69.9)*
Fast food	161 (22.2)*	181 (18.5)	142 (14.9)	96 (20.3)
Sweets	267 (36.8)	378 (38.6)	348 (36.5)	187 (39.6)*

*- the highest percentage of affirmative answers for individual types of diet/eating habits

In the study group of 3155 people, 84 respondents did not report information about their diets, and in 3071 participants, a large variation in terms of diet was found. Most respondents declared that they based their diet on warm soups (69.6%), silage (53.8%), and fruits and vegetables (46.9%). Respondents were allowed to give several answers in a single question, e.g. give an answer about the amount of meat eaten and at the same time about the frequency of eating soup. A significant proportion of survey participants (47.1%) supplement their diet with the recommended amount of water above eight glasses per day. Groups were also distinguished in terms of the amount of meat consumed: the largest group were people who admitted that their diet consists of a large amount of meat (30.6%), people eating small amounts of meat constituted a smaller number of respondents (25.3%), and the smallest group consisted of vegetarians (2.1%). Consumption of processed and high-calorie products, such as fast food, was reported admitted to 18.5% of respondents, and 37.6% to eating sweets. A low-calorie diet was reported by 20.76%, while a high-calorie diet was chosen by 18.3%. The effect of diet on the duration of COVID-19 symptoms was analyzed. Based on the data obtained, it was found that a high-calorie diet was associated with shorter symptoms of the disease (1–5 days), or their complete absence. High calories and shorter symptoms of COVID-19 disease were also confirmed in the group of respondents who admitted to eating large amounts of fast food. A similar relationship occurred in people who consumed large amounts of meat. Limiting the amount of meat in the diet in the next group of subjects was associated with longer symptoms that often lasted more than 10 days. Hydration of the body also had a significant impact on the course of the disease. Drinking a large amount of water was reported mainly by people, in whom the symptoms usually lasted from 1 to 5 days.

### The impact of physical activity on the course of COVID-19

**Table 4:** The impact of physical activity on the duration of COVID 19 symptoms

Physical activity	1–5 Days	6–10 Days	> 10 Days	No symptoms
Less than once a month *N* = 367 N(%)	74 (20.2)	103 (28.1)	144 (39.2)*	46 (12.5)
Once a month *N* = 224 N(%)	51 (22.8)	62 (27.7)	70(31.3)*	41(18.3)
Several times a month *N* = 627 N(%)	149 (23.8)	221 (35.3)*	175 (27.9)	82 (31.1)
Once a week *N* = 394 N(%)	81 (20.6)	142 (36.2)*	124 (31.5)	47 (11.9)
Several times a week *N* = 860 N(%)	213 (24.8)*	269 (31.3)	251 (29.2)	127 (14.8)
Every day *N* = 654 N(%)	153(23.4)	184 (28.1)	187 (28.6)	130 (19.9)*

*- the highest percentage of study participants depending on the duration of symptoms

As part of the questionnaire, study participants subjectively assessed their physical activity. Of the 3126 people who answered the question about physical activity 52.0% considered their physical activity to be good, average was reported by 23.1%, very good by 19.3%, and low 5.5%. The survey showed that the most respondents performed physical activity several times a week (27.5%), slightly fewer respondents admitted to daily activities (20.9%), a similar group of respondents declared several times a month (20.1%), smaller groups were people who reported that they play sports once a week (12.6%) and less than once a month (11.8%). The smallest group consisted of people who reported physical activity once a month (7.2%). Participants who rated their activity at a lower level (less than a few times a month) tended to remain sick longer, over 10 days. An inverse relationship was observed in people who were active more than once a week. Respondents in this group had symptoms lasting less than 10 days. Daily physical activity was reported by a significant number of respondents (20.9%) and resulted in the absence of symptoms of the disease or their significant shortening (up to five days). The results allow us to conclude that undertaking physical activity could has a positive effect on shortening the time of COVID-19 disease.

## Discussion

The body’s resistance to pathogenic infections is associated with many factors. These factors include stress exposure, amount of sleep, physical stress, environmental conditions and diet. Proper nutrition and the supply of vitamins, macro- and microelements are of great importance in maintaining the body in good condition and have a key role in metabolic processes and immunological. The well-known vitamin C supplementation prevents frequent infections by supporting the immune system. This approach forced us to analyze the impact of food consumption on the duration of infection, the number of symptoms, and the course of the disease itself [23].

The aim of our work was to analyze the impact of factors such as diet, physical activity and smoking on the course of COVID-19 infection and the length of symptoms. The dietary results show the possible effect of the food consumed on the duration of the symptoms of COVID-19 disease or the complete absence of symptoms. The results include statistical analyses of questionnaires collected from patients with positive RT-PCR results, and they show that diet could has a significant impact on the appearance of symptoms and their duration. Patients who ate a balanced diet reported the least symptoms of all the types of diets analyzed. Patients who ate a lot of meat were less likely to complain of symptoms, and an especially statistically significant result turned out to be the absence of headaches and lack of taste. Patients who consumed small amounts of meat reported headaches, runny nose, cough, skin rashes or sore throats more often, a result that was statistically significant. A balanced diet is usually rich in ingredients that support the immune system. A special role may be played in this case by vitamins of group A, C, D, and E, which are an important aspect of the regulation of the immune system. Retinol is responsible for maintaining the proper continuity of mucous membranes because it limits the possibility of microorganism-associated breakage of this barrier. Vitamin A deficiency leads to weakening of the phagocytic properties of macrophages and causes a reduction the number of T lymphocytes. Cholecalciferol interacts with receptors on the cells of the immune system and regulates the specific and non-specific responses of the body in addition to enhancing the phagocytic and chemotactic properties of macrophages [24, 25]. Consumption of ascorbic acid has a positive effect on the stimulation of prostaglandin production, cytokine production, and elimination of the immunosuppressive effect of histamine [26]. The work of the immune system can also be stimulated by consuming the right amount of fats. Scientific studies have shown the effects of fatty acids on regulating the production of prostaglandins and cytokines. The consequence of such an impact is a reduction in the appearance of inflammation. In addition, a high content of fatty acids can have antiemetic and neuroprotective effects. Such properties may be associated with the absence of headaches, throat pains, and/or loss of smell or taste in people following a diet model rich in fats [27]. Vegetarians were less likely to complain of back pain or headaches. In people whose diet was based mainly on fresh fruits and vegetables, several symptoms were more common; these included runny nose, cough, cold sweats, headaches, lack of appetite, muscle and throat pains, diarrhea. The frequent presence of these symptoms may be the result of a consumption of a reduced amount of polyunsaturated fats. On the basis of the research, it was shown that a high-energy diet had an effect on symptom reduction or a complete absence of symptoms. Patients were less likely to report headaches, coughs, or runny noses, symptoms characteristic of the common cold. Similarly, diet can had an impact on the duration of COVID-19 symptoms. Patients on a balanced diet, those with heavy meat, or those consuming high-calorie and fast foods reported a shorter duration of symptoms. Patients on a low-calorie diet that was poor in vitamins, proteins and fats reported longer persistence of symptoms and were more likely to complain of headaches, runny noses or coughs. In addition, they often reported the occurrence of muscle or back pain. An important aspect of the diet during infection with the SARS-CoV-2 virus is the consumption of the right amount of water. A large amount of water above eight glasses per day could had the effect of shortening the duration of symptoms.

A correlation analysis was performed between cigarette smoking and the symptoms of COVID-19 disease. Symptoms, such as cough, fever and chills, headache, weakness, sore throat, and diarrhea were more common in the group of smokers. In the study group with active smokers, the highest percentage of people who did not develop symptoms was observed (22.9%), and the symptoms were present for a much shorter time period (1–5 days) than in the other study groups. In an Italian multicenter case-control study, Meini et al. showed an unexpectedly low incidence of symptoms in smokers among patients hospitalized with COVID-19. Some studies have shown a relative increase in the risk of severe disease and death in smoking patients compared to non-smokers [28–33]. On the other hand, evidence in which a smaller percentage of people hospitalized with COVID-19 occurs among smokers exists. It can therefore be suggested that the chemical compounds contained in cigarettes offer protection against infection with the SARS-CoV-2 virus [33, 34]. However, it should be remembered that smoking undoubtedly has a detrimental effect on the respiratory and cardiovascular systems [19]. This finding prompts the question: Are smokers really at a lower risk of sars-CoV-2 infection due to the protective effects of nicotine? The data require further confirmation among a larger group of respondents. However, this discovery may become a stimulus for the creation of new hypotheses about individual predispositions or possible strategies aimed at reducing the risk of SARS-CoV-2 infection.

As the results of the research taking up physical activity is positively correlated with a reduction in the duration of COVID 19 symptoms. The analysis of questionnaires completed by participants of the study conducted in the United Kingdom Biobank confirms the relationship between lack of physical activity and sedentary lifestyle and more frequent occurrence of infection. Among the 387,109 cases studied, those who did not engage in physical activity were 1.51 times more likely to develop COVID-19 in relation to the reference sample. Physical activity, even at a relatively low level, has been shown to have a protective effect against infection with the SARS-CoV-2 virus. Those participating in less than 150 min of moderate or vigorous exercise, which is still less than the minimum accepted by the researchers, were less likely to get sick [35]. Evidence that a sedentary lifestyle is harmful to health has been published [19]. Regular exercise performed throughout an individual’s life results in a longer period of health and delays the onset of more than 40 different chronic diseases [36]. People who exercise more than the currently recommended minimum level of activity are less likely to develop breast cancer, colorectal cancer, diabetes, coronary heart disease, or stroke [37]. As it has been proven, exercise activates the immune system, positively affecting the mobilization of natural killer (NK) cells and supporting chemotaxis and neutrophil phagocytosis. This process also responsible for modulating macrophages present in adipose tissue, activated by inflammation. During intense exercise, the proliferation and migration of CD4 + and CD8 + T cells also intensifies [38, 39]. The reduction in the duration of COVID-19 symptoms may also be due to the more effective spread of the virus throughout the body. An earlier start of the stage of multiplication of the SARS-CoV-2 virus and the presence of the virus in many host cells may result in a more intense course of the disease and thus a faster reaction of the cells of the immune system. One of the potential mechanisms of the spread of the SARS-CoV-2 virus may be the transport of virus particles using elements of the host cell cytoskeleton, such as actin or myosin II. The virus interacts with phylopodia after which it enters the cell, in a way that resembles surfing [40]. People who undertake physical activity have greater heart efficiency and a higher value of maximum oxygen utilization (VO2 max) than inactive people. They also have a larger lung volume and different muscle fiber composition [41]. The SARS-CoV-2 virus has been proven to attach to the cells that are precursors of red blood cells [42]. Greater lung volume and better heart performance may result in the fact that when the body inhales air into the lungs, it also inhales a larger amount of virus particles, which can then penetrate the cells present in the bloodstream that are precursors of red blood cells. Infected cells can be transported through the circulatory system to the whole body and by inducing inflammation, can activate the immune system’s response to fight an ongoing infection. Effective heart performance can also drive the immune system by transporting cells and molecules that are components of the immune system to the site of infection. All such mechanisms would then lead to a shorter course of the disease and consequently, a shortening of the time of occurrence of symptoms related to the COVID-19 disease. In summary, the results suggest that lifestyle parameters, including diet, frequency of physical activity, and smoking, have a significant impact on the risk of infection and the duration of symptoms of COVID-19 disease.

## Conclusions

All such mechanisms would then lead to a shorter course of the disease and consequently, a shortening of the time of occurrence of symptoms related to the COVID-19 disease. In summary, the results suggest that lifestyle parameters, including diet, frequency of physical activity, and smoking, have a significant impact on the risk of infection and the duration of symptoms of COVID-19 disease.

## Data Availability

The data that support the findings of this study are not openly available due to reasons of sensitivity and are available from the corresponding author upon reasonable request. Data are located in controlled access data storage at Regional Sciences and Technology Center.

## Abbreviations

ACE: Angiotensin Converting Receptor
RCNT: Regional Sciences and Technology Center
